# Supplementary material for: Studies on the Enantioselective Synthesis of E-Ethylidene-bearing Spiro[indolizidine-1,3′-oxindole] Alkaloids
Source: Molecules. 2021 Jan 15;26(2):428. doi: 10.3390/molecules26020428 (PMC7829768; doi:10.3390/molecules26020428)
Supplement: Supplementary file 1 [file molecules-26-00428-s001.pdf]

Studies on the Enantioselective Synthesis of *E*-Ethylidene-Bearing Spiro[indolizidine-1,3′-oxindole] Alkaloids

Nihan Yayik, Maria Pérez, Elies Molins, Joan Bosch, and Mercedes Amat*

Supporting Information Available

I) Copies of ^{1}H and ^{13}C NMR spectra pages: S2-S17

II) X- ray crystallographic data for compounds **4** pages S18-S23

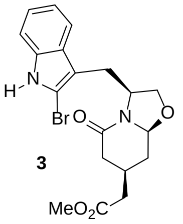

3

(400 MHz, CDCl3)

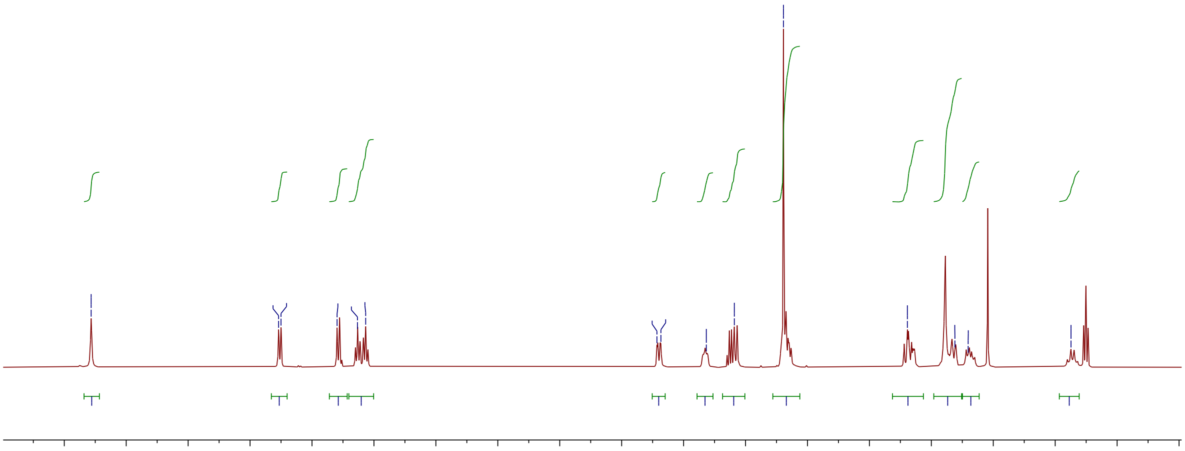

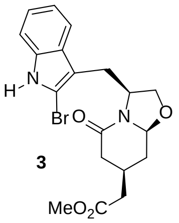

3

(100.6 MHz, CDCl3)

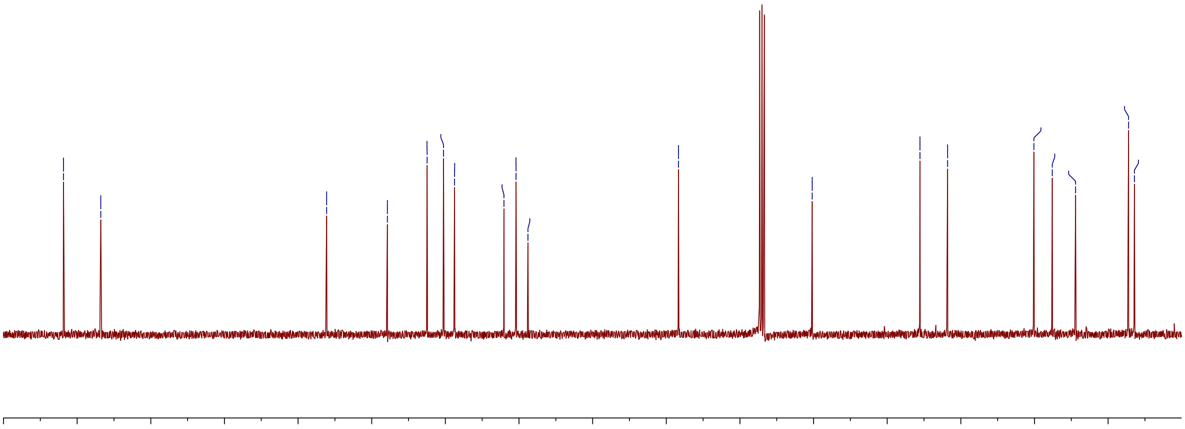

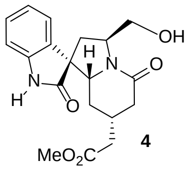

4

(400 MHz, CDCl3)

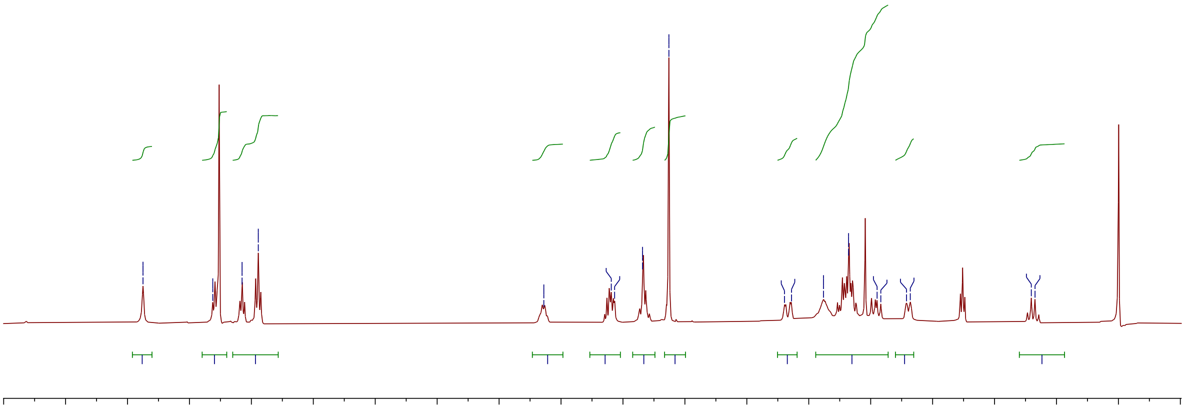

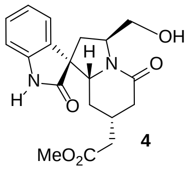

4

(100.6 MHz, CDCl3)

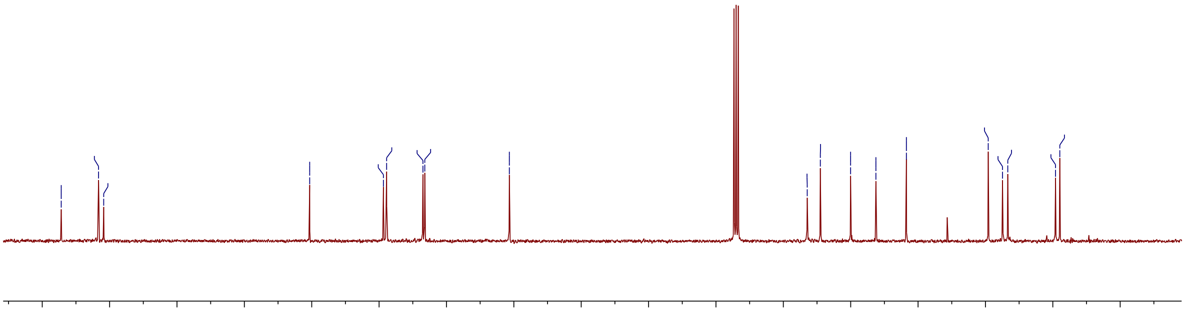

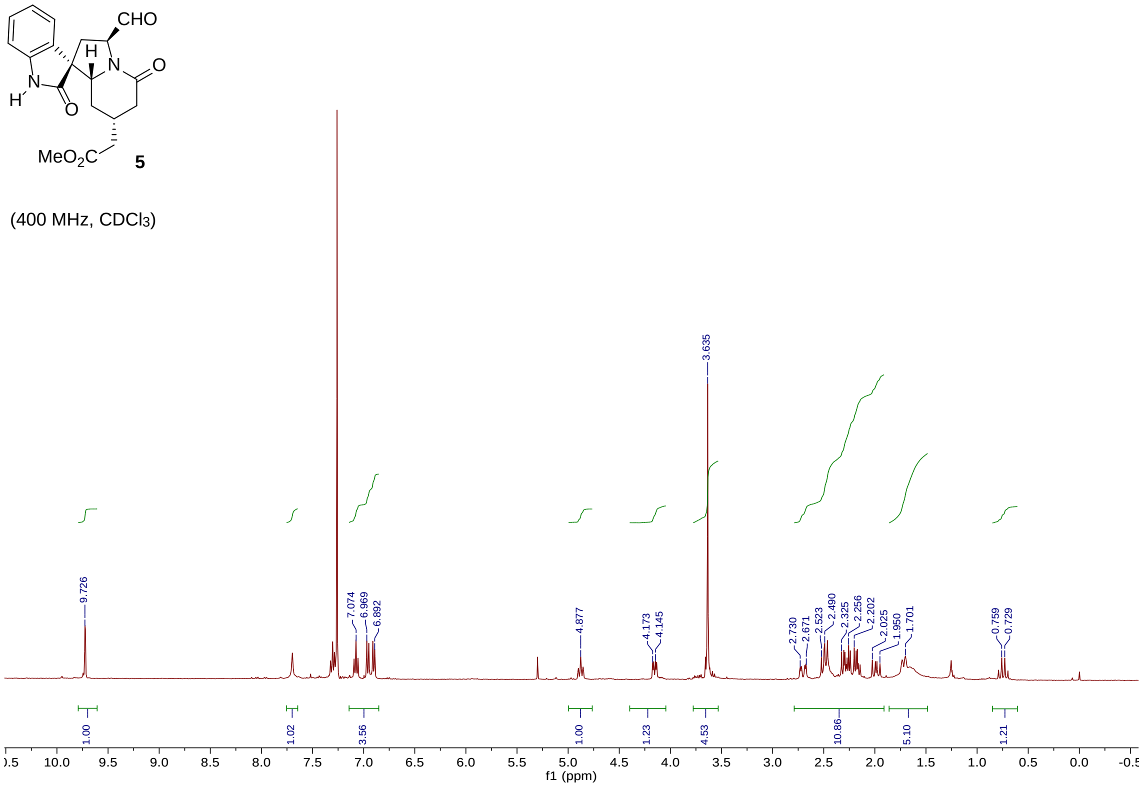
CHO
H
N
O
H
N
O
MeO2C
5
(400 MHz, CDCl3)
f1 (ppm)

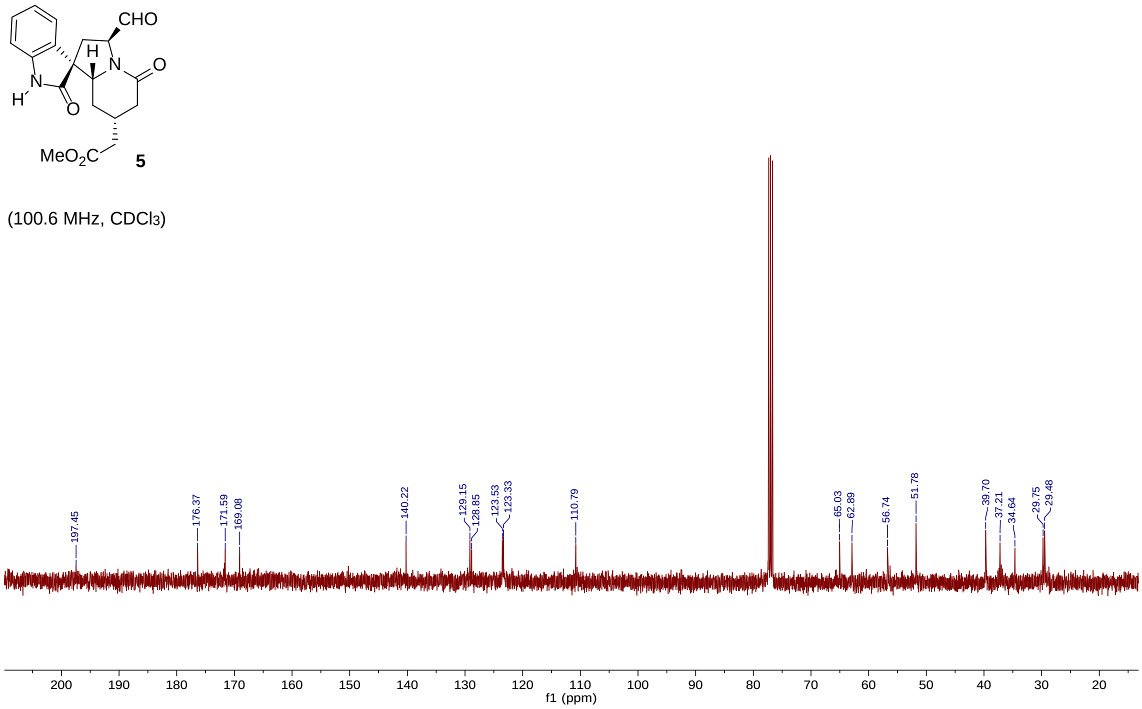
CHO
H
N
O
H
N
O
MeO2C
5
(100.6 MHz, CDCl3)
f1 (ppm)

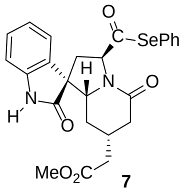

7

(400 MHz, $CDCl_3$)

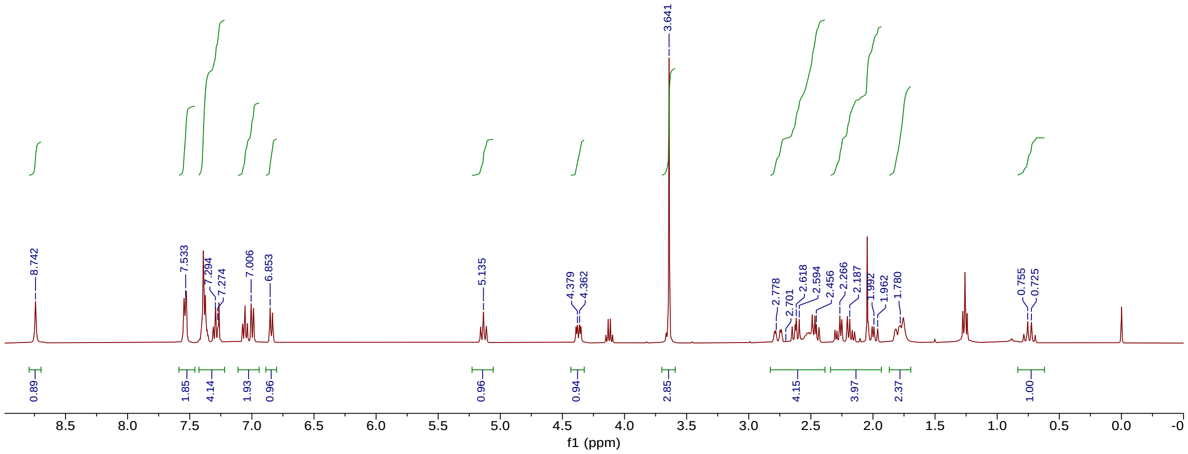

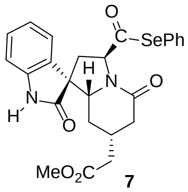

7

(100.6 MHz, $CDCl_3$)

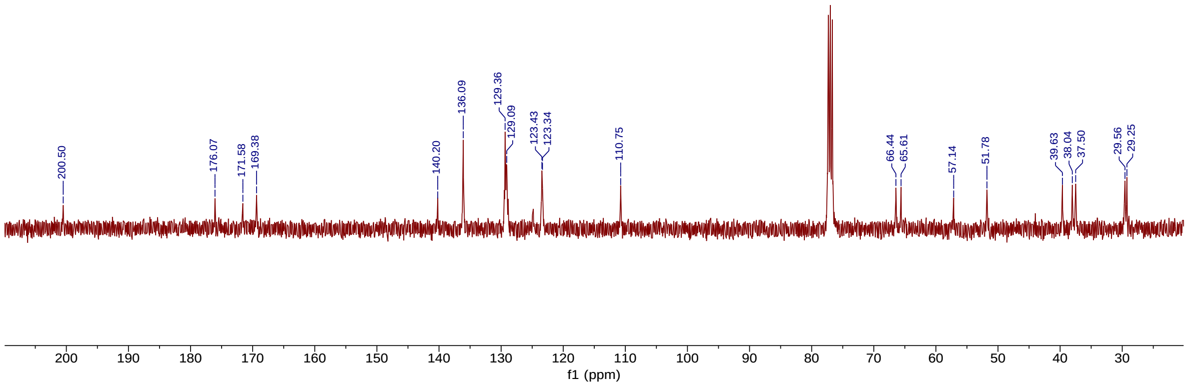

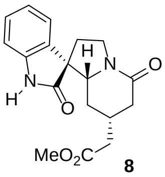

8

(400 MHz, $CDCl_3$)

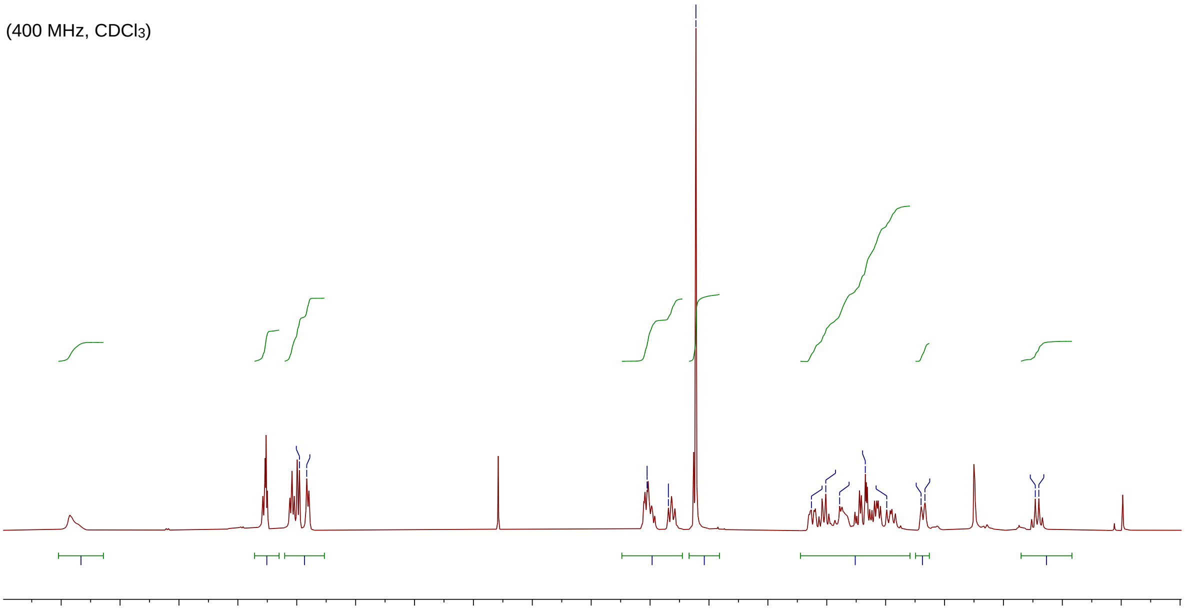

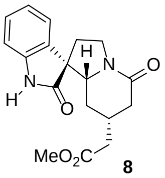

8

(100.6 MHz, $CDCl_3$)

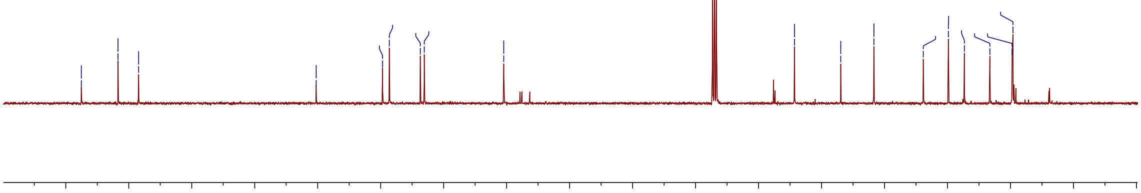

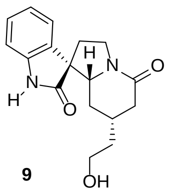

9

(400 MHz, $CDCl_3$)

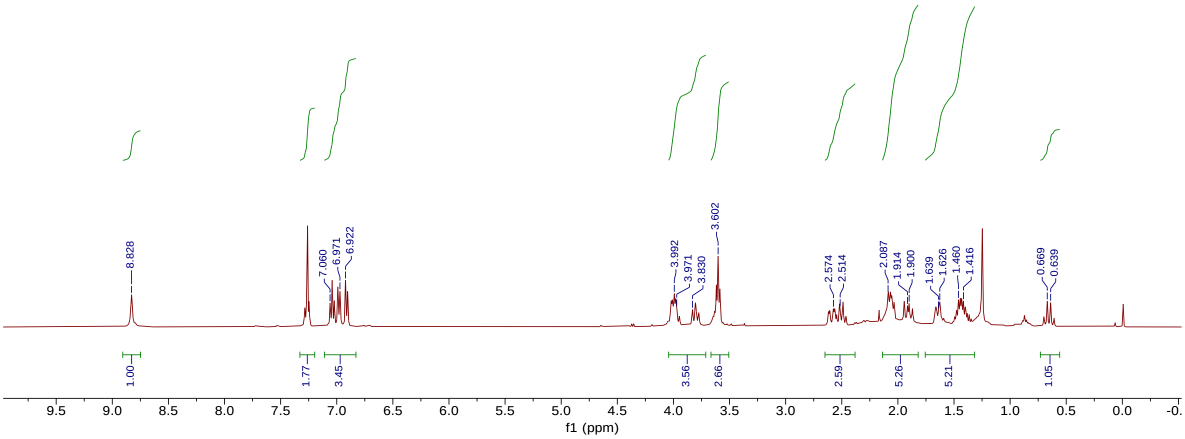

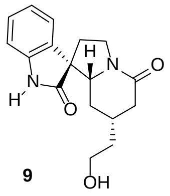

9

(100.6 MHz, $CDCl_3$)

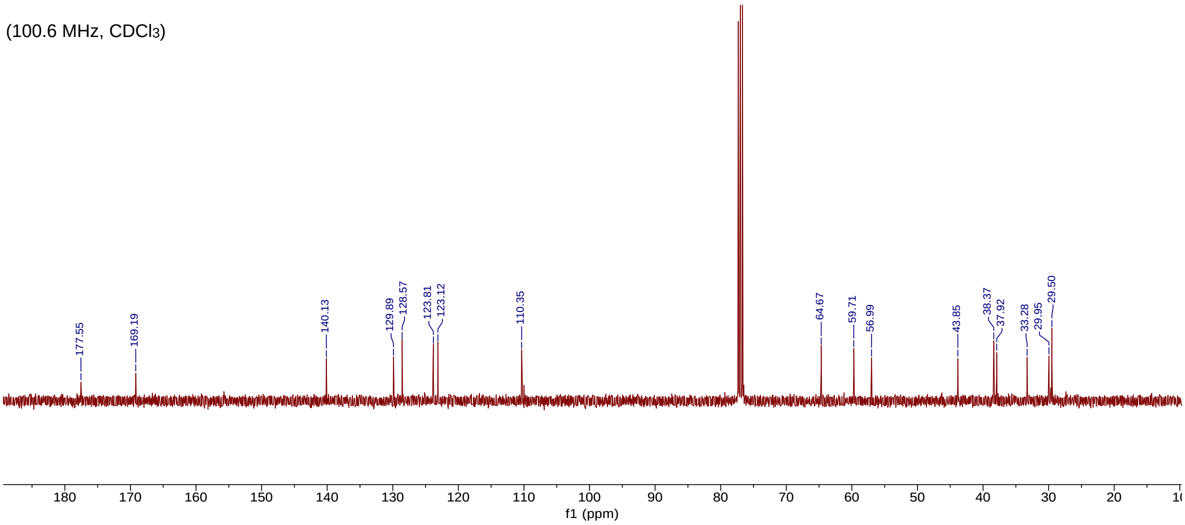

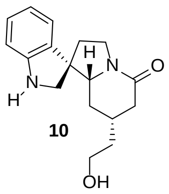

10

(400 MHz, $CDCl_3$)

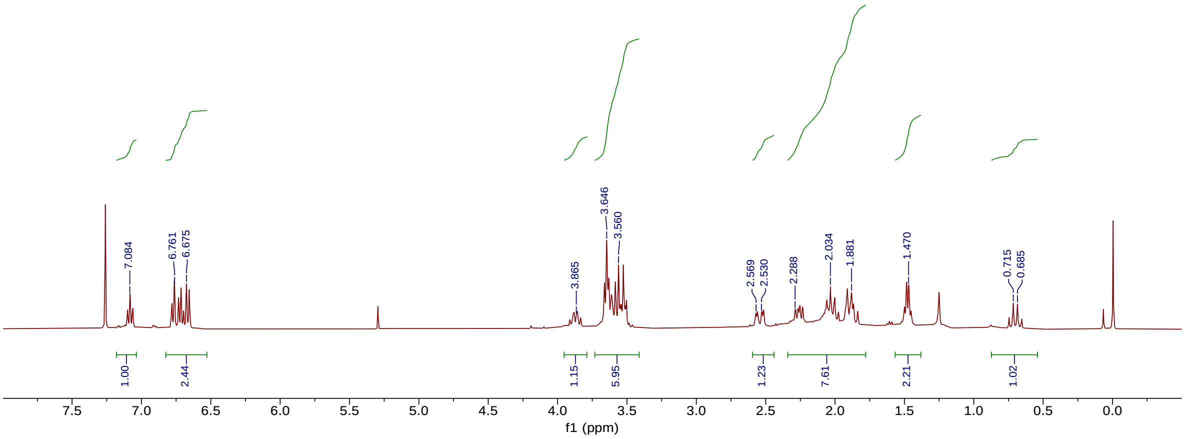

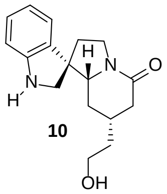

10

(100.6 MHz, $CDCl_3$)

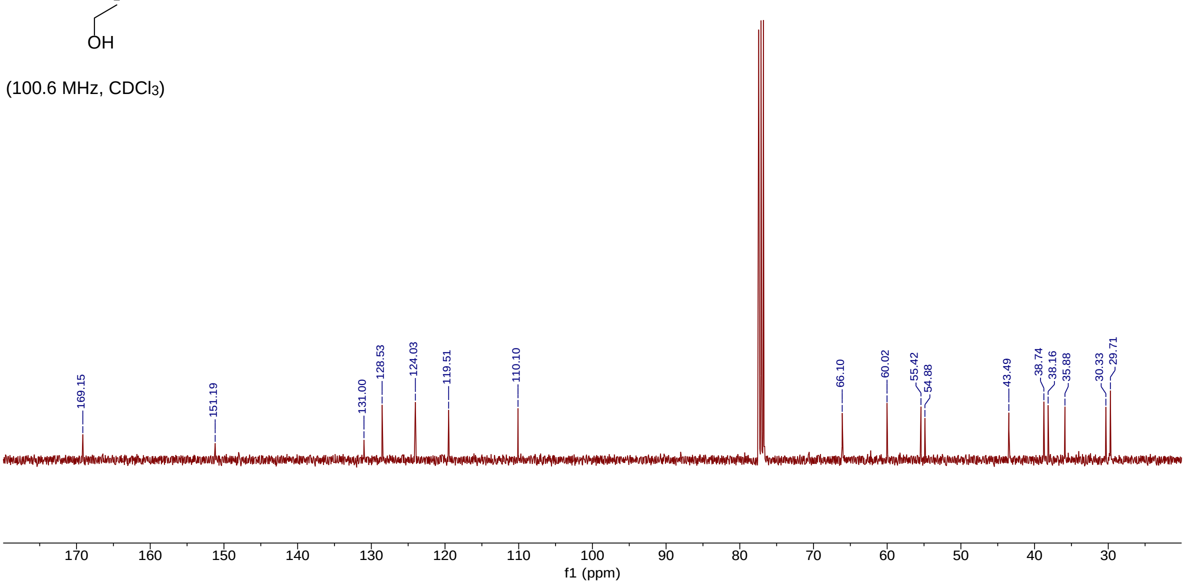

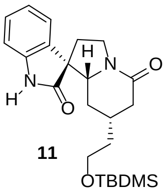

11

(400 MHz, $CDCl_3$)

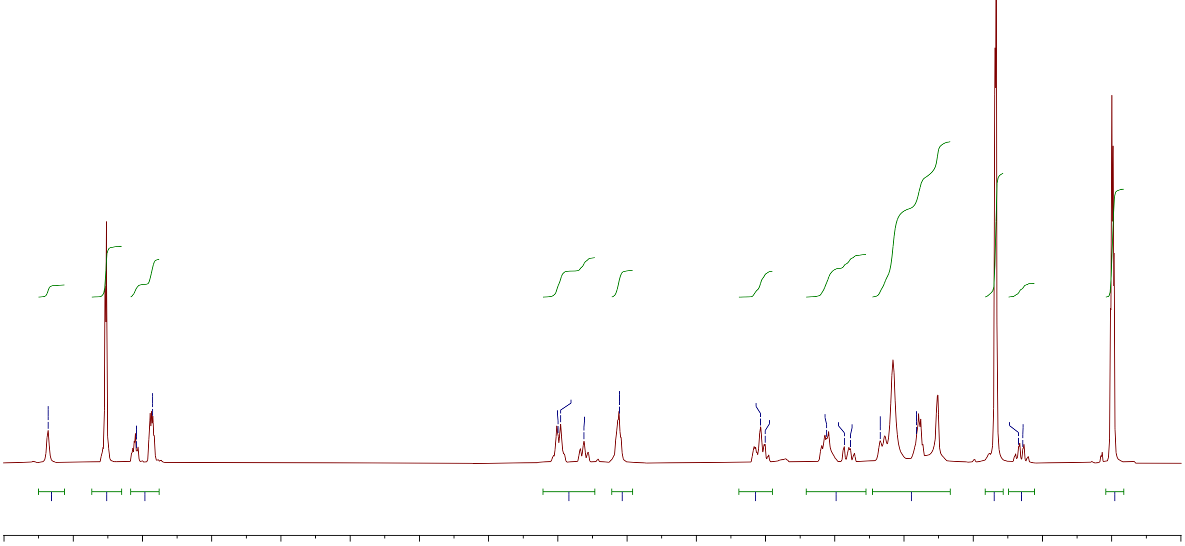

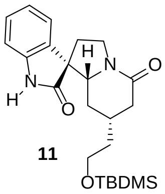

11

(100.6 MHz, $CDCl_3$)

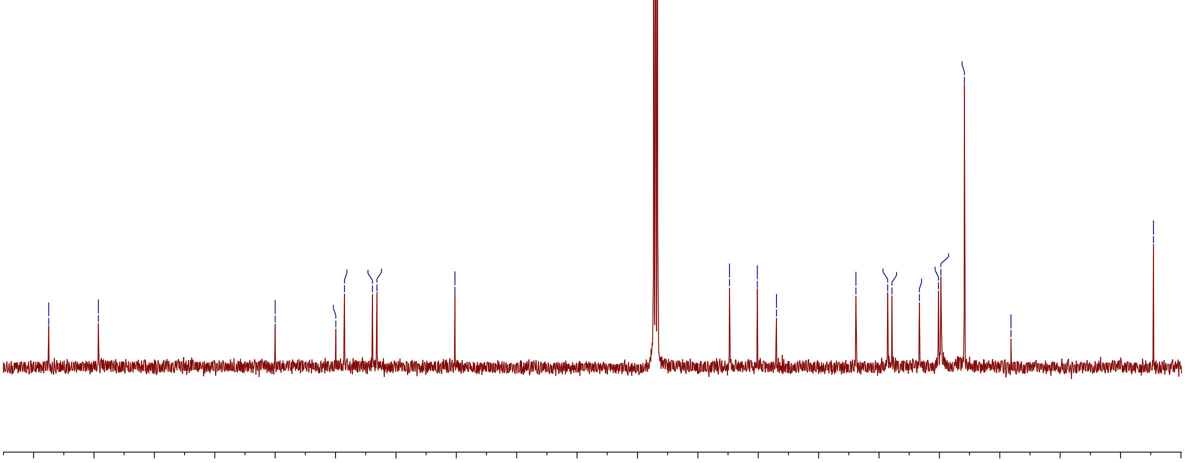

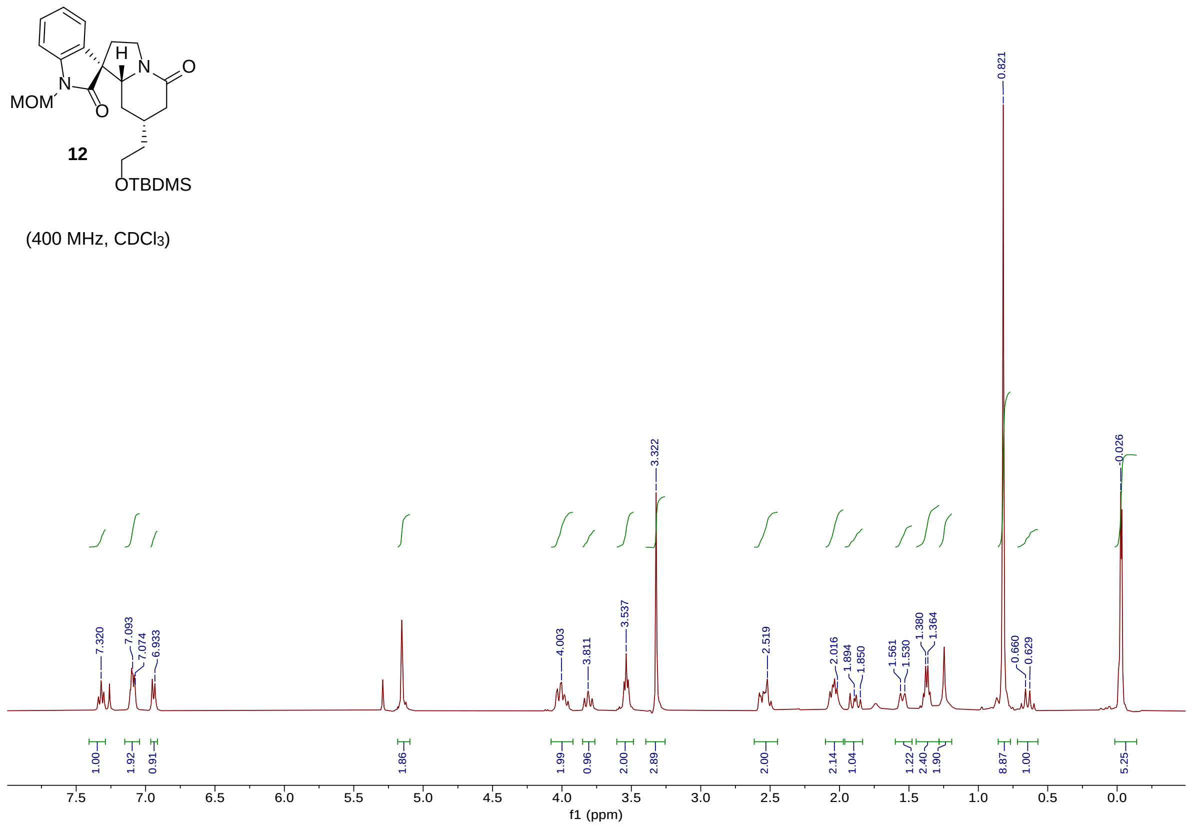

12

(400 MHz, $CDCl_3$)

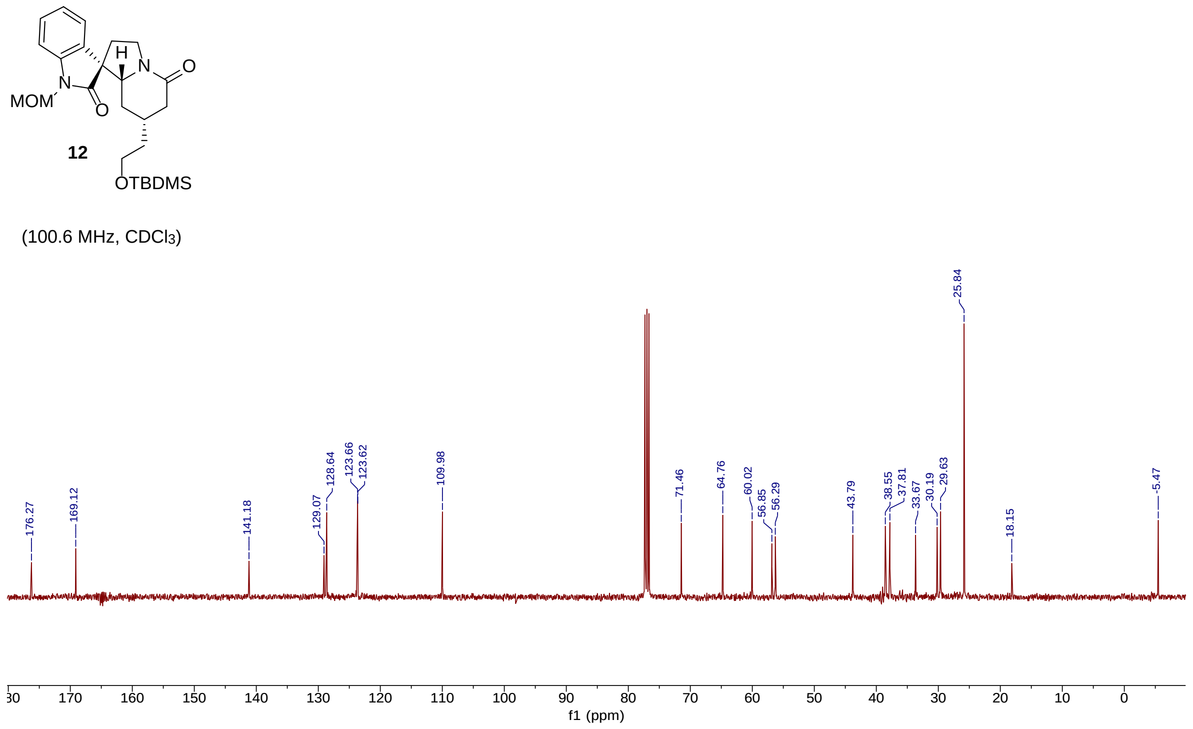

12

(100.6 MHz, $CDCl_3$)

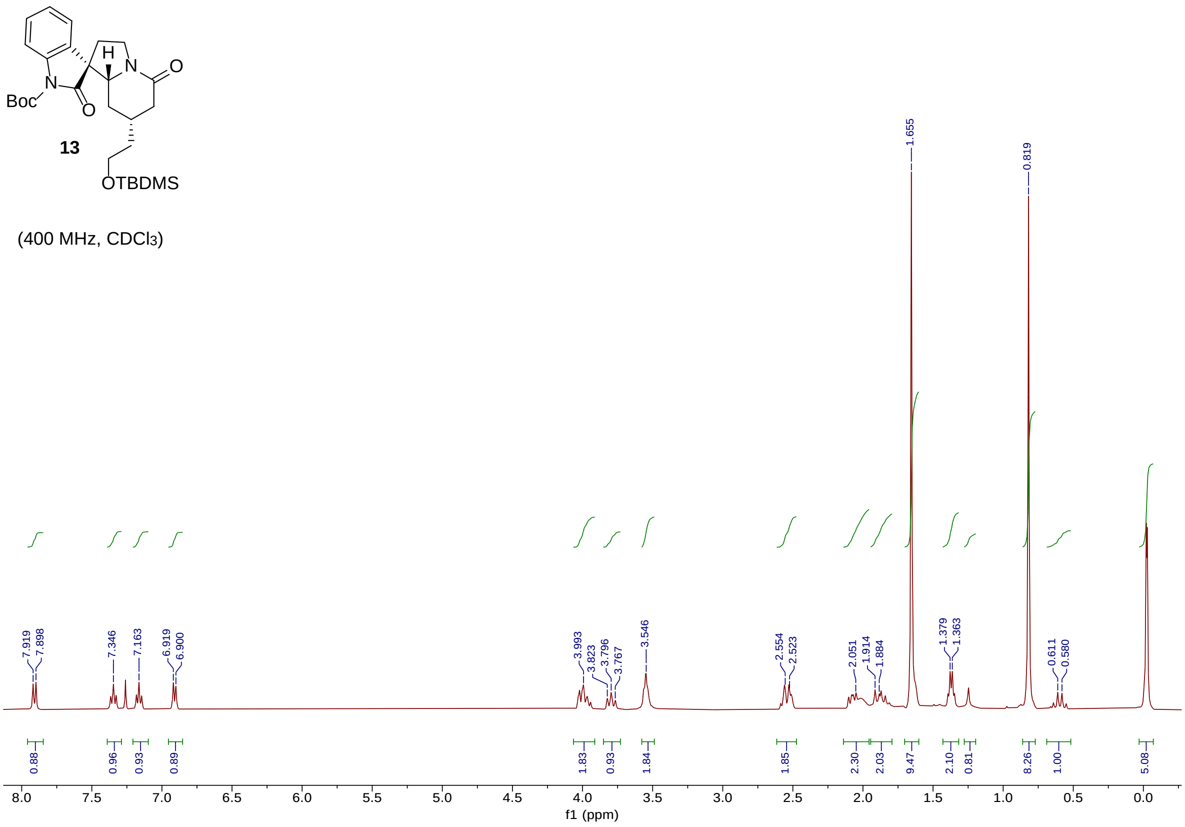

Boc
13
OTBDMS
(400 MHz, CDCl3)
7.919 7.898 7.346 7.163 6.919 6.900 3.993 3.823 3.796 3.767 3.546 2.554 2.523 2.051 1.914 1.884 1.655 1.379 1.363 0.819 0.611 0.580
0.88 0.96 0.93 0.89 1.83 0.93 1.84 1.85 2.30 2.03 9.47 2.10 0.81 8.26 1.00 5.08
f1 (ppm)

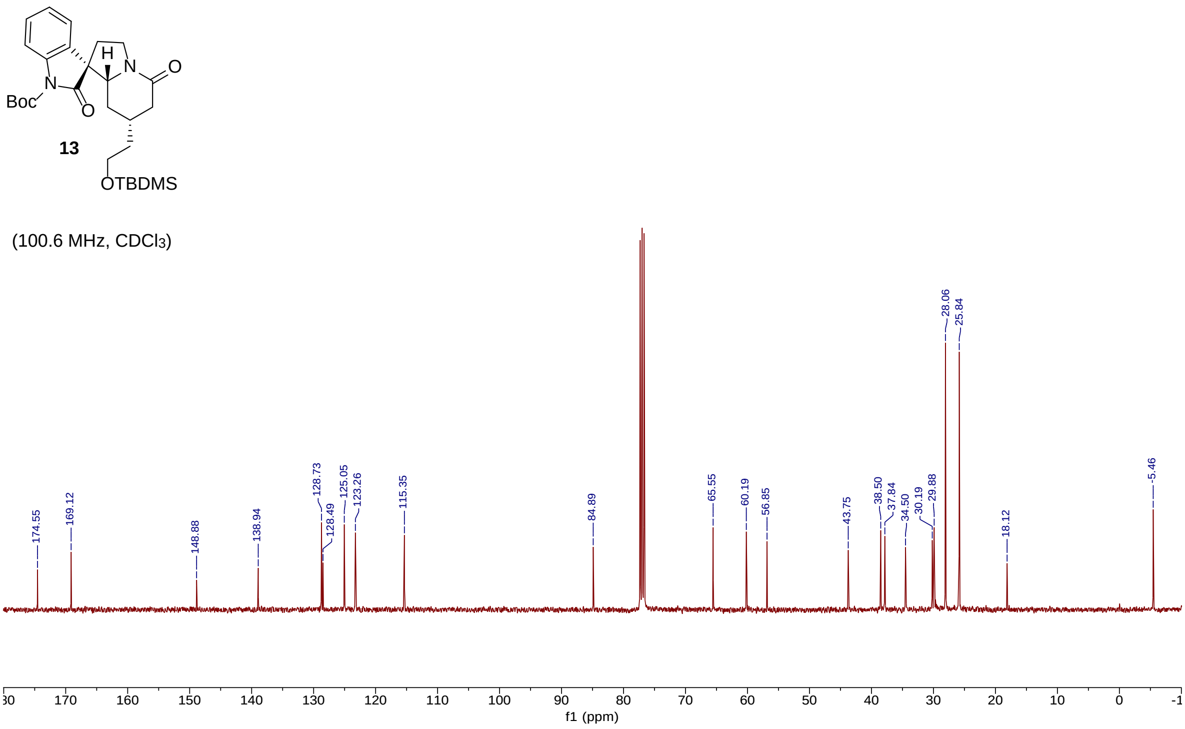

Boc
13
OTBDMS
(100.6 MHz, CDCl3)
174.55 169.12 148.88 138.94 128.73 128.49 125.05 123.26 115.35 84.89 65.55 60.19 56.85 43.75 38.50 37.84 34.50 30.19 29.88 28.06 25.84 18.12 -5.46
f1 (ppm)

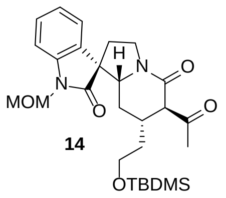

14

(400 MHz, $CDCl_3$)

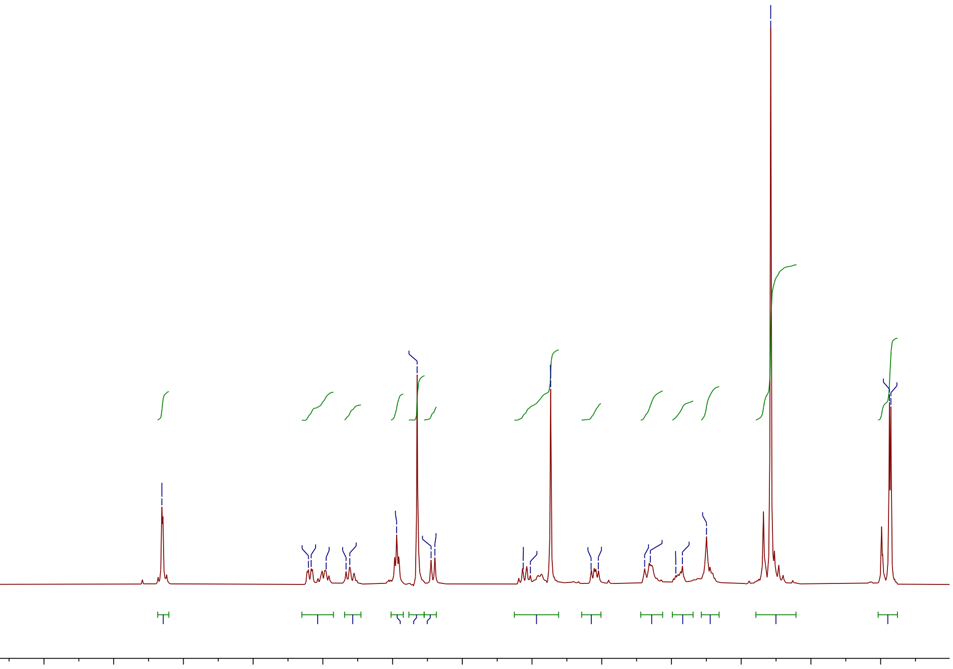

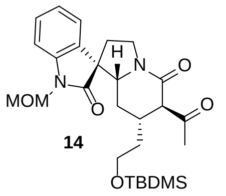

14

(100.6 MHz, $CDCl_3$)

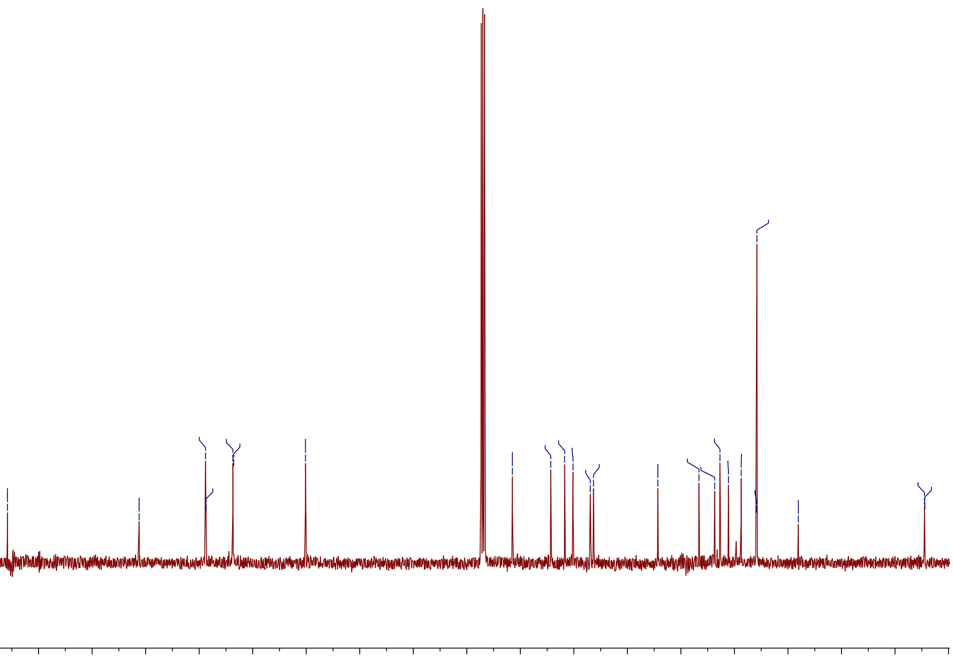

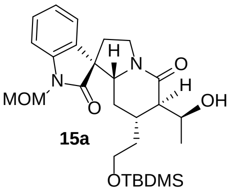

15a

(400 MHz, $CDCl_3$)

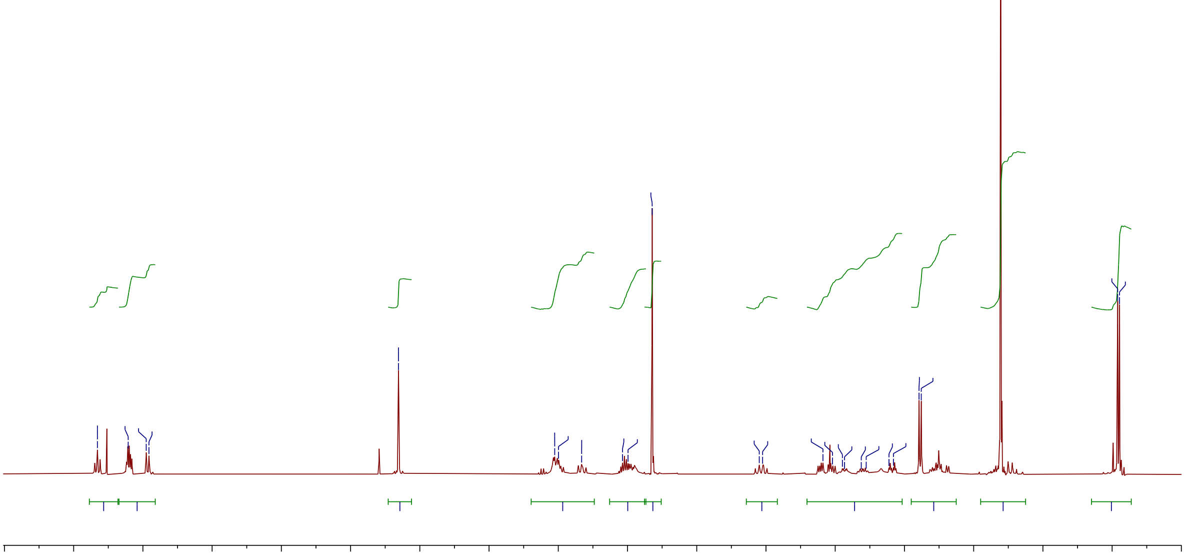

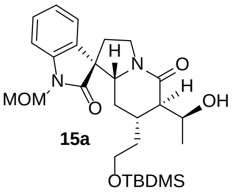

15a

(100.6 MHz, $CDCl_3$)

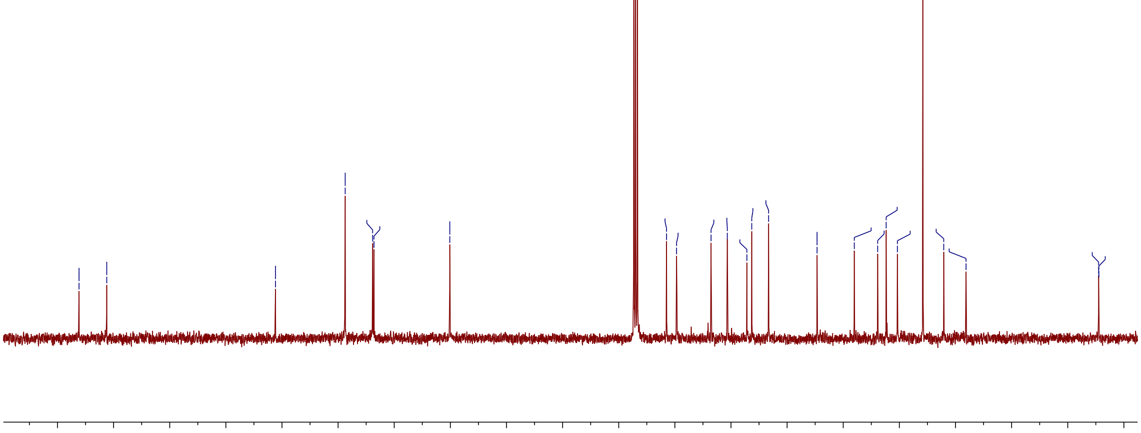

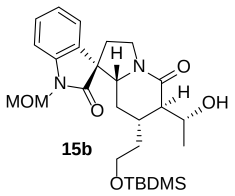

(400 MHz, $CDCl_3$)

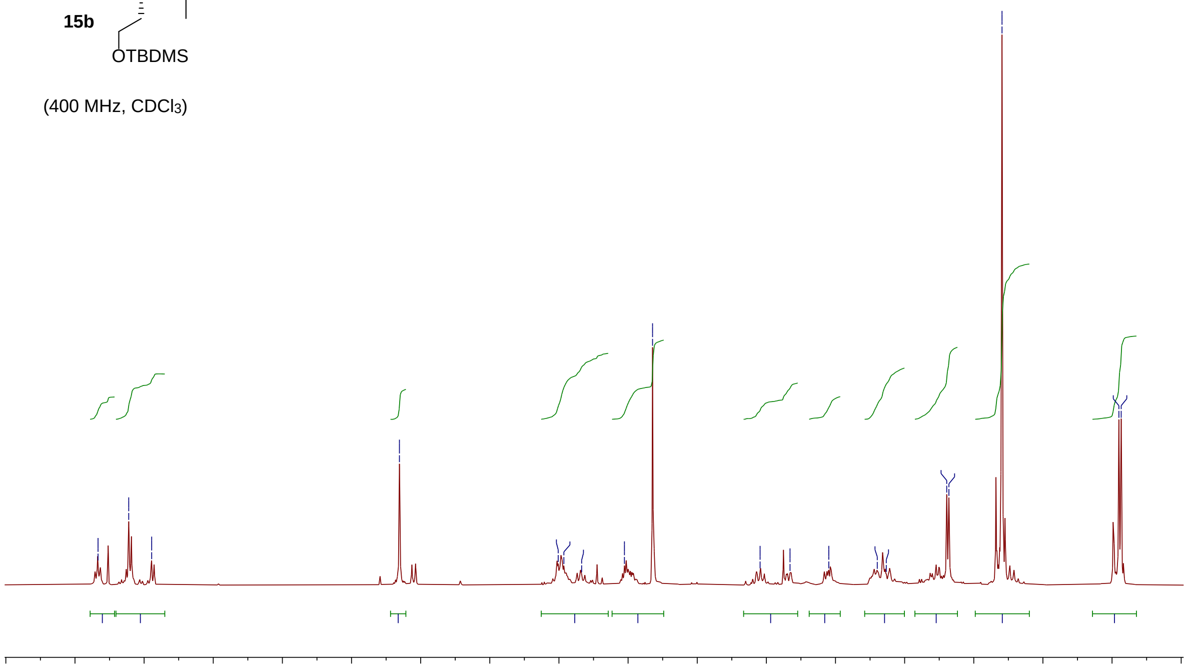

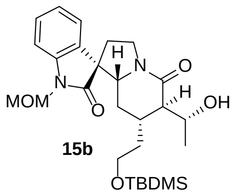

(100.6 MHz, $CDCl_3$)

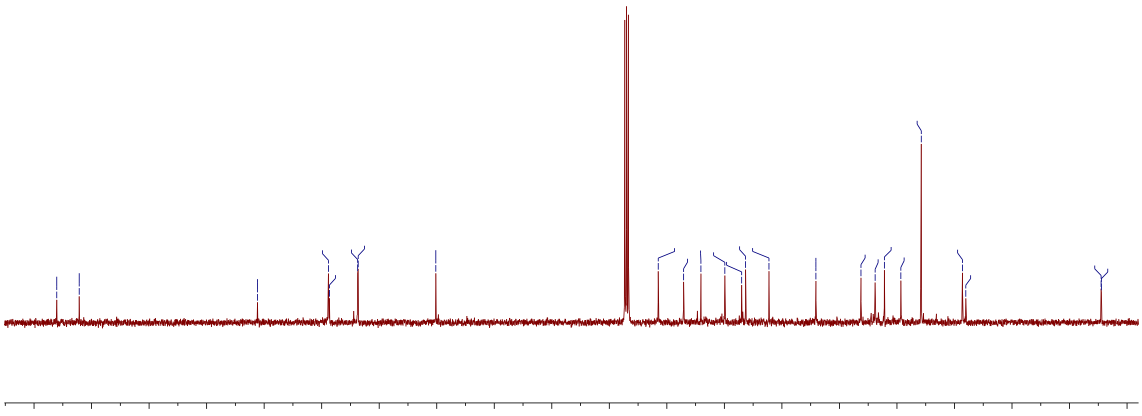

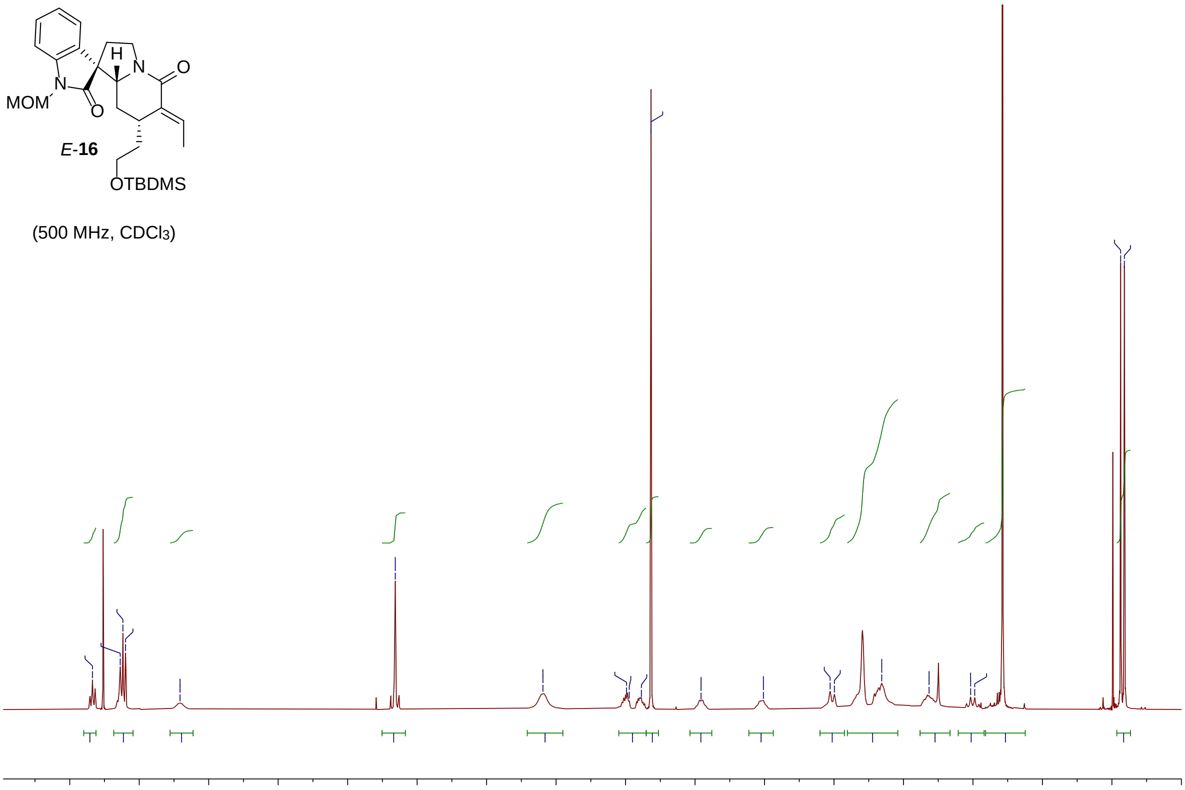
H
N
O
MOM
N
O
E-16
OTBDMS
(500 MHz, CDCl3)

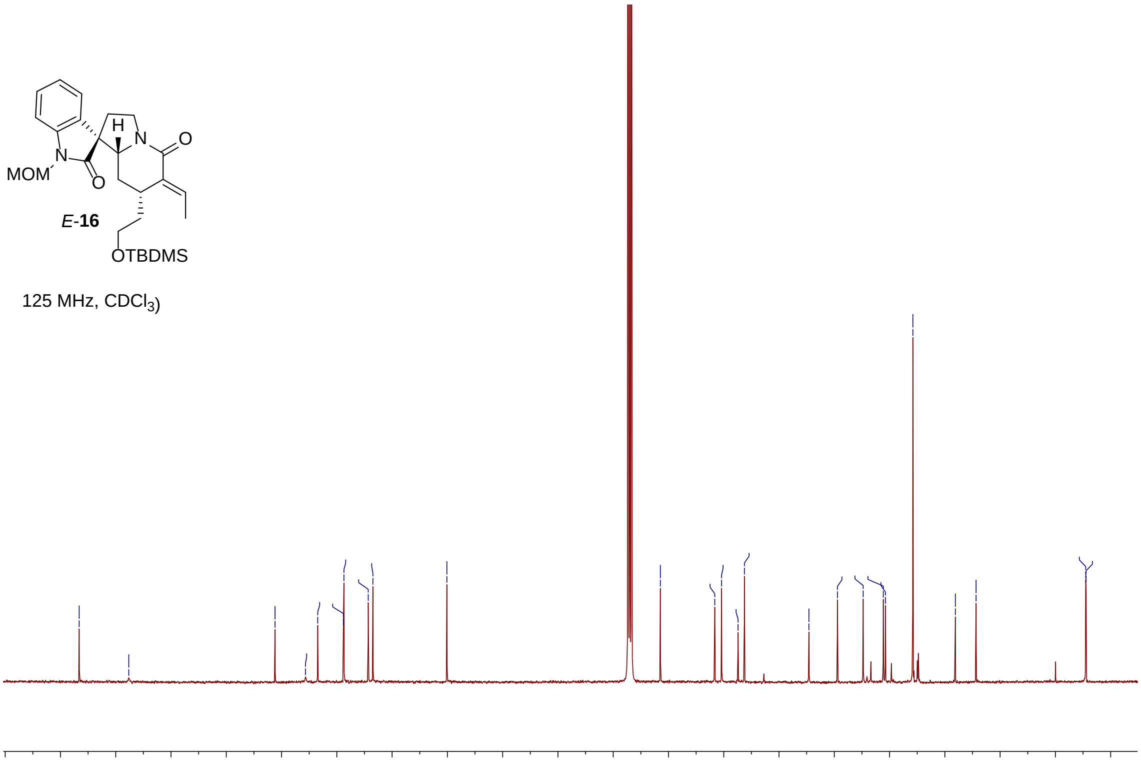
H
N
O
MOM
N
O
E-16
OTBDMS
125 MHz, CDCl3)

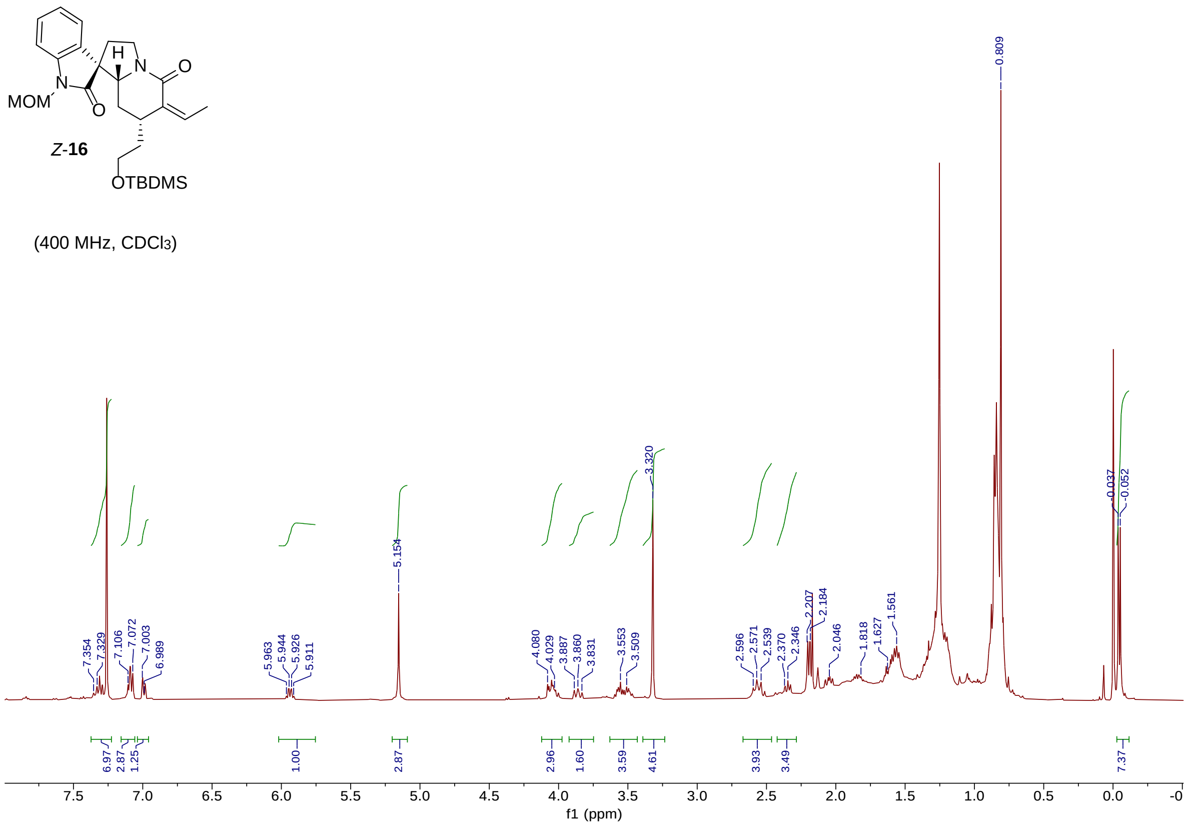
MOM
Z-16
OTBDMS
(400 MHz, CDCl3)
f1 (ppm)

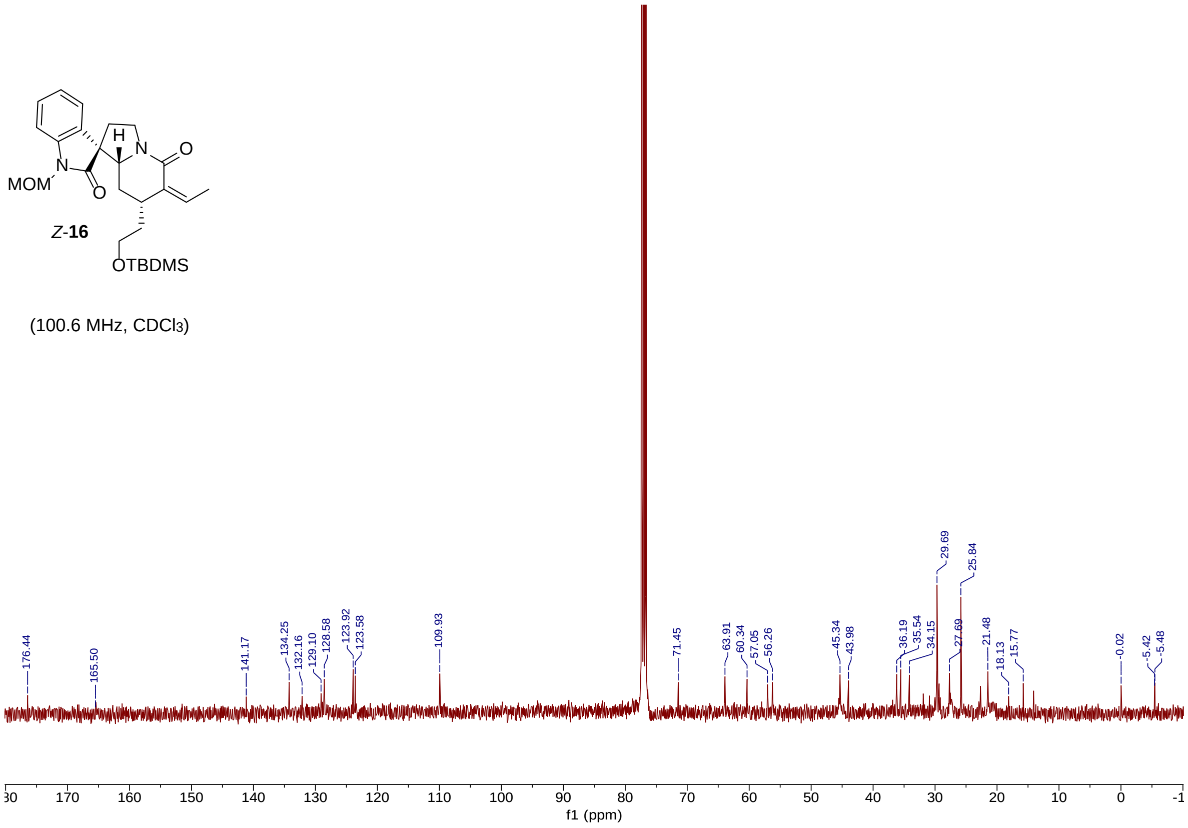
MOM
Z-16
OTBDMS
(100.6 MHz, CDCl3)
f1 (ppm)

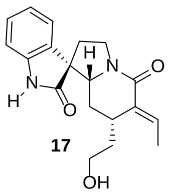

17

(400 MHz, $CDCl_3$)

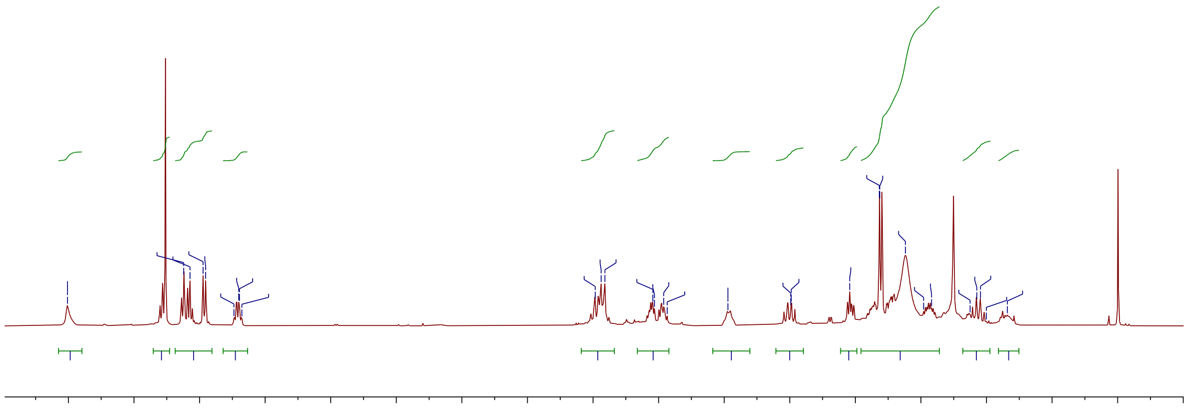

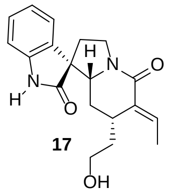

17

(100.6 MHz, $CDCl_3$)

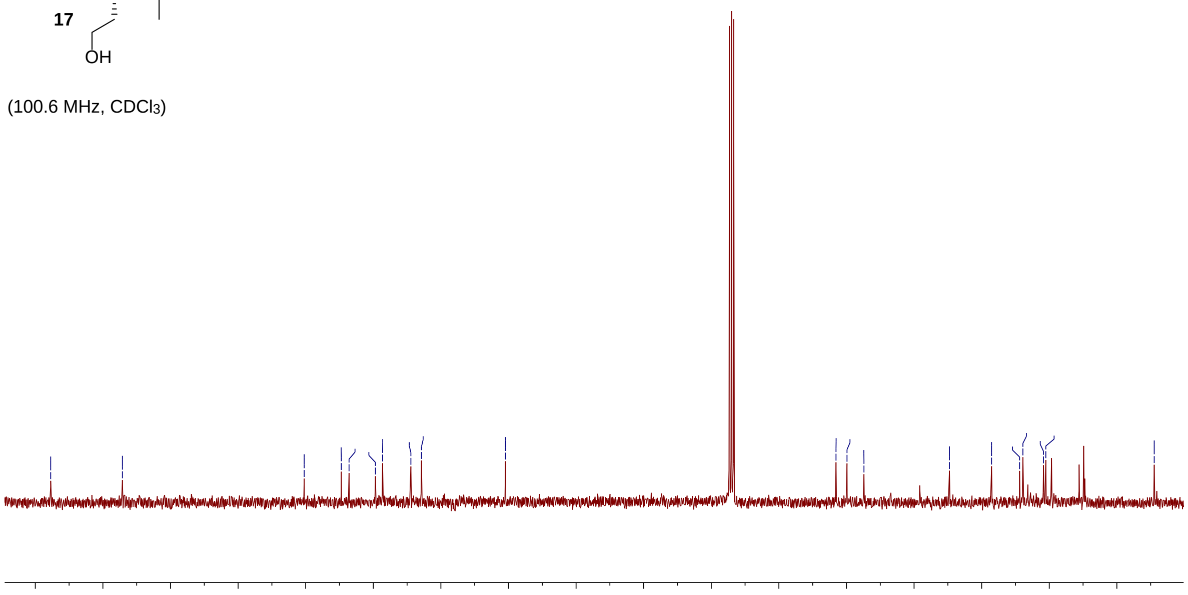

Table 1. Crystal data and structure refinement for **4**.

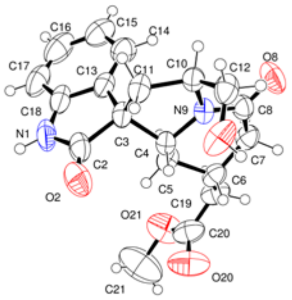

Identification code	Jb153	
Empirical formula	C19 H22 N2 O5	
Formula weight	358.38	
Temperature	294(2) K	
Wavelength	0.71073 Å	
Crystal system	Orthorhombic	
Space group	P 21 21 21	
Unit cell dimensions	a = 6.6025(3) Å	α= 90°.
	b = 15.2412(6) Å	β= 90°.
	c = 17.8186(7) Å	γ = 90°.
Volume	1793.09(13) $Å^3$	
Z	4	
Density (calculated)	1.328 Mg/m^3	
Absorption coefficient	0.097 mm^{-1}	
F(000)	760	
Crystal size	0.280 x 0.180 x 0.150 mm^3	
Theta range for data collection	1.758 to 28.390°.	
Index ranges	-8<=h<=8, -20<=k<=20, -23<=l<=23	
Reflections collected	52832	
Independent reflections	4481 [R(int) = 0.0708]	
Completeness to theta = 25.242°	100.0 %	
Refinement method	Full-matrix least-squares on F^2	
Data / restraints / parameters	4481 / 0 / 237	
Goodness-of-fit on F^2	1.013	
Final R indices [I>2sigma(I)]	R1 = 0.0525, wR2 = 0.1286	
R indices (all data)	R1 = 0.0823, wR2 = 0.1482	
Absolute structure parameter	-0.2(6)	
Largest diff. peak and hole	0.535 and -0.141 e.$Å^{-3}$	

Table 2. Atomic coordinates (x 10^4) and equivalent isotropic displacement parameters ($Å^2$x 10^3) for **4**. U(eq) is defined as one third of the trace of the orthogonalized U^{ij} tensor.

	x	y	z	U(eq)
O(2)	3488(5)	4488(2)	7505(2)	82(1)
O(8)	9293(4)	3079(2)	4872(1)	76(1)
O(12)	5846(5)	2119(2)	6502(1)	71(1)
O(20)	826(5)	5303(3)	4440(2)	113(1)
O(21)	2376(5)	6549(2)	4207(2)	102(1)
N(1)	5352(6)	5768(2)	7481(2)	70(1)
N(9)	7348(4)	3712(2)	5747(1)	44(1)
C(2)	4936(7)	4923(3)	7303(2)	59(1)
C(3)	6638(5)	4602(2)	6782(2)	46(1)
C(4)	5776(5)	4308(2)	6013(2)	42(1)
C(5)	5299(6)	5015(2)	5441(2)	49(1)
C(6)	4924(5)	4596(2)	4679(2)	50(1)
C(7)	6822(5)	4112(2)	4436(2)	55(1)
C(8)	7895(5)	3592(2)	5030(2)	51(1)
C(10)	8383(5)	3268(2)	6368(2)	48(1)
C(11)	7613(6)	3759(2)	7065(2)	53(1)
C(12)	7919(6)	2294(2)	6383(2)	59(1)
C(13)	8001(5)	5389(2)	6737(2)	50(1)
C(14)	9801(6)	5530(2)	6368(2)	64(1)
C(15)	10702(7)	6359(3)	6398(3)	77(1)
C(16)	9780(9)	7023(3)	6795(3)	88(2)
C(17)	8001(9)	6895(3)	7184(3)	80(1)
C(18)	7136(7)	6063(2)	7155(2)	61(1)
C(19)	4297(6)	5283(3)	4092(2)	65(1)
C(20)	2316(7)	5699(3)	4261(2)	71(1)
C(21)	459(11)	7021(5)	4313(4)	149(3)

Table 3. Bond lengths [Å] and angles [°] for **4**.

O(2)-C(2)	1.218(5)
O(8)-C(8)	1.242(4)
O(12)-C(12)	1.411(5)
O(20)-C(20)	1.198(5)
O(21)-C(20)	1.299(6)
O(21)-C(21)	1.468(7)
N(1)-C(2)	1.355(5)
N(1)-C(18)	1.388(6)
N(9)-C(8)	1.340(4)
N(9)-C(4)	1.459(4)
N(9)-C(10)	1.467(4)
C(2)-C(3)	1.537(5)
C(3)-C(13)	1.501(5)
C(3)-C(11)	1.523(4)
C(3)-C(4)	1.551(4)
C(4)-C(5)	1.516(4)
C(5)-C(6)	1.522(4)
C(6)-C(7)	1.516(5)
C(6)-C(19)	1.536(5)
C(7)-C(8)	1.500(5)
C(10)-C(12)	1.515(5)
C(10)-C(11)	1.535(4)
C(13)-C(14)	1.375(5)
C(13)-C(18)	1.391(5)
C(14)-C(15)	1.398(5)
C(15)-C(16)	1.375(7)
C(16)-C(17)	1.378(7)
C(17)-C(18)	1.392(6)
C(19)-C(20)	1.485(6)
C(20)-O(21)-C(21)	116.9(4)
C(2)-N(1)-C(18)	112.4(3)
C(8)-N(9)-C(4)	125.9(3)
C(8)-N(9)-C(10)	122.1(3)
C(4)-N(9)-C(10)	111.9(2)
O(2)-C(2)-N(1)	127.4(4)
O(2)-C(2)-C(3)	125.4(3)

N(1)-C(2)-C(3)	107.2(3)
C(13)-C(3)-C(11)	116.0(3)
C(13)-C(3)-C(2)	102.5(3)
C(11)-C(3)-C(2)	112.2(3)
C(13)-C(3)-C(4)	113.8(2)
C(11)-C(3)-C(4)	101.7(2)
C(2)-C(3)-C(4)	110.9(3)
N(9)-C(4)-C(5)	111.8(2)
N(9)-C(4)-C(3)	101.9(2)
C(5)-C(4)-C(3)	117.7(2)
C(4)-C(5)-C(6)	109.6(3)
C(7)-C(6)-C(5)	108.9(3)
C(7)-C(6)-C(19)	111.1(3)
C(5)-C(6)-C(19)	111.4(3)
C(8)-C(7)-C(6)	116.5(3)
O(8)-C(8)-N(9)	120.2(3)
O(8)-C(8)-C(7)	121.6(3)
N(9)-C(8)-C(7)	118.2(3)
N(9)-C(10)-C(12)	111.8(3)
N(9)-C(10)-C(11)	103.4(2)
C(12)-C(10)-C(11)	113.4(3)
C(3)-C(11)-C(10)	106.5(2)
O(12)-C(12)-C(10)	112.6(3)
C(14)-C(13)-C(18)	119.7(3)
C(14)-C(13)-C(3)	132.0(3)
C(18)-C(13)-C(3)	108.4(3)
C(13)-C(14)-C(15)	119.4(4)
C(16)-C(15)-C(14)	119.7(5)
C(15)-C(16)-C(17)	122.1(4)
C(16)-C(17)-C(18)	117.4(4)
N(1)-C(18)-C(13)	109.5(3)
N(1)-C(18)-C(17)	128.8(4)
C(13)-C(18)-C(17)	121.6(4)
C(20)-C(19)-C(6)	113.0(3)
O(20)-C(20)-O(21)	123.2(5)
O(20)-C(20)-C(19)	124.2(4)
O(21)-C(20)-C(19)	112.6(4)

Table 4. Anisotropic displacement parameters (Å^{2}x 10^3) for **4**. The anisotropic displacement factor exponent takes the form: $-2\pi^2$[$h^2a^{*2}U^{11}$ + ... + 2 h k a* b* U^{12}]

	U^{11}	U^{22}	U^{33}	U^{23}	U^{13}	U^{12}
O(2)	88(2)	98(2)	60(2)	-3(2)	26(2)	7(2)
O(8)	80(2)	89(2)	59(2)	-8(1)	23(1)	25(2)
O(12)	104(2)	62(2)	48(1)	13(1)	-15(1)	-31(2)
O(20)	63(2)	127(3)	148(3)	35(3)	-2(2)	-19(2)
O(21)	89(2)	87(2)	129(3)	37(2)	18(2)	6(2)
N(1)	91(3)	69(2)	49(2)	-23(2)	-3(2)	27(2)
N(9)	56(2)	39(1)	37(1)	-4(1)	4(1)	-1(1)
C(2)	75(2)	65(2)	36(2)	-5(1)	3(2)	15(2)
C(3)	59(2)	43(2)	36(2)	-7(1)	-1(1)	4(2)
C(4)	49(2)	40(1)	37(1)	-5(1)	2(1)	-1(1)
C(5)	61(2)	44(2)	43(2)	0(1)	-7(1)	-3(2)
C(6)	54(2)	56(2)	40(2)	3(1)	-3(1)	-14(2)
C(7)	59(2)	71(2)	37(2)	-2(2)	4(1)	-13(2)
C(8)	53(2)	55(2)	45(2)	-8(1)	10(1)	-3(2)
C(10)	50(2)	47(2)	47(2)	-8(1)	-4(2)	5(1)
C(11)	74(2)	43(2)	43(2)	-4(1)	-8(2)	5(2)
C(12)	81(3)	43(2)	52(2)	-4(1)	-14(2)	12(2)
C(13)	64(2)	44(2)	43(2)	-7(1)	-14(2)	5(2)
C(14)	64(2)	51(2)	76(2)	-7(2)	-7(2)	-4(2)
C(15)	81(3)	66(2)	85(3)	1(2)	-20(2)	-17(2)
C(16)	115(4)	53(2)	95(3)	-6(2)	-40(3)	-13(3)
C(17)	114(4)	50(2)	77(3)	-23(2)	-36(3)	13(2)
C(18)	86(3)	51(2)	44(2)	-12(1)	-18(2)	15(2)
C(19)	62(2)	83(3)	50(2)	17(2)	-9(2)	-12(2)
C(20)	63(2)	91(3)	60(2)	24(2)	-15(2)	-16(2)
C(21)	124(5)	152(6)	171(7)	62(5)	36(5)	56(5)

Table 5. Hydrogen bonds for **4** [Å and °].

D-H...A	d(D-H)	d(H...A)	d(D...A)	<(DHA)
O(12)-H(12)...O(8)#1	0.82	1.86	2.671(3)	173.0
N(1)-H(1)...O(12)#2	0.86	2.07	2.854(4)	152.0
C(7)-H(7B)...O(20)#3	0.97	2.36	3.206(5)	145.4
C(12)-H(12A)...O(8)	0.97	2.52	3.083(5)	116.9

Symmetry transformations used to generate equivalent atoms:

#1 x-1/2,-y+1/2,-z+1 #2 -x+1,y+1/2,-z+3/2 #3 x+1,y,z
